# Fresh Air: A Necessity

**Published:** 1882-11

**Authors:** 


### FRESH AIR: A NECESSITY.

A Dr. Oswald writes:—

The Cold-Air Fallacy.—The influence of anti-naturalism is most strikingly illustrated in our superstitious dread of fresh air. The air of the out-door world, of the woods and hills, is, *par excellence*, a product of Nature—of wild, free and untamable Nature—and therefore the presumptive sources of innumerable evils. Cold air is the general scapegoat of all sinners against Nature. When the knee-joints of the young debauchee begin to weaken, he suspects that he has "taken cold." If an old glutton has a cramp in his stomach, he ascribes it to an incautious exposure on coming home from a late supper. Toothache is supposed to result from "draughts"; croup, neuralgia, mumps, etc., from the "raw March wind." When children have to be forced to sleep in unventilated bedrooms till their lungs putrefy with their own exhalations, the *materfamilias* reproaches herself with the most sensible thing she has been doing for the last hundred nights—"opening the windows last August when the air was so stiflingly hot." The old dyspeptic, with his cupboard full of patent nostrums, can honestly acquit himself of having yielded to any natural impulse; after sweltering all summer behind hermetically closed windows, wearing flannel in the dog-days, abstaining from cold water when his stomach craved it, swallowing drugs till his appetite has given way to chronic nausea, his conscience bears witness that he has done what he could to suppress the original depravity of Nature; only once the enemy got a chance at him: in rummaging his garret for a warming-pan he stood half a minute before a broken window—to that half-minute, accordingly, he attributes his rheumatism. For catarrh there is a stereotyped explanation: "Catched cold." That settles it. The invalid is quite sure that her cough came on an hour after returning from that sleigh-ride. She felt a pain in her chest the moment her brother opened that window. There is no doubt of it—it is all the night-air's fault.

The truth is, that cold-air often reveals the existence of a disease. It initiates the reconstructive process, and thus apparently the disease itself, but there is a wide difference between a proximate and an original cause. A man can be too *tired* to sleep and too *weak* to be sick. Bleeding, for the time being, may "break up" an inflammatory disease; the system must regain some little strength before it can resume the work of reconstruction. The vital energy of a person breathing the stagnant air of an unventilated stove-room is often inadequate to the task of undertaking a restorative process—though the respiratory organs, clogged with phlegm and all kinds of impurities, may be sadly in need of relief. But, during a sleigh-ride, or a few hours' sleep before a window left open by accident, the bracing influence of the fresh air revives the drooping vitality, and nature avails herself of the chance to begin repairs, the lungs reveal their diseased condition, *i. e.*, they proceed to rid themselves of the accumulated impurities. Persistent in-door life would have aggravated the evil by postponing the crisis, or by turning a temporary affection into a chronic disease. But in a plurality of cases Nature will seize even upon a transient improvement of external circumstances: a cold night that disinfects the atmosphere of the bedroom in spite of closed windows, a draught of cool air from an adjoining room, or one of those accidental exposures to wind and weather which the veriest slave of the cold-air superstition cannot always avoid. For, rightly understood, the external symptoms of disease constitute a restorative process that cannot be brought to a satisfactory issue till the cause of the evil is removed. So that, in fact, the air-hater confounds the cause of his recovery with the cause of his disease. Among nations who pass their lives out-doors, catarrh and scrofula are almost unknown; not fresh air, but the want of it, is the cause of countless diseases, of fatal diseases where people are in the habit of *nailing down* their windows every winter to keep their children from opening them. "In one such den," says Dr. Bock, "I was so overcome with nausea that I could not speak till I had knocked out a pane of glass. That is about the best thing one could do in most sick-rooms"—except knocking out the whole window. The only objection to a "draught" through a defective window is, that the draught is generally not strong enough. An influx of fresh air into a fusty sick-room is a ray of light into darkness, a messenger of Vishnu visiting an abode of the damned. Cold is a disinfectant and under the pressure of a high wind a modicum of oxygen will penetrate a house in spite of closed windows. This circumstance alone has preserved the lives of thousands whom no cough-syrup or cod-liver oil could have saved.—*Popular Science Monthly*.

